# Raw Garlic Consumption and Risk of Liver Cancer: A Population-Based Case-Control Study in Eastern China

**DOI:** 10.3390/nu11092038

**Published:** 2019-08-31

**Authors:** Xing Liu, Aileen Baecker, Ming Wu, Jin-Yi Zhou, Jie Yang, Ren-Qiang Han, Pei-Hua Wang, Ai-Min Liu, Xiaoping Gu, Xiao-Feng Zhang, Xu-Shan Wang, Ming Su, Xu Hu, Zheng Sun, Gang Li, Zi-Yi Jin, Su Yon Jung, Lina Mu, Na He, Qing-Yi Lu, Liming Li, Jin-Kou Zhao, Zuo-Feng Zhang

**Affiliations:** 1 Department of Epidemiology, Fielding School of Public Health, University of California, Los Angeles (UCLA), CA 90095, USA; 2 Department of Epidemiology, School of Public Health, Fudan University, Shanghai 200032, China; 3 Department of Nutrition, Harvard T.H. Chan School of Public Health, Boston, MA 02115, USA; 4 Jiangsu Provincial Center for Disease Control and Prevention, Nanjing 210009, China; 5 Dafeng Center for Disease Control and Prevention, Dafeng 224100, China; 6 Ganyu Center for Disease Control and Prevention, Ganyu 222003, China; 7 Chuzhou County Center for Disease Control and Prevention, Chuzhou 223001, China; 8 Tongshan County Center for Disease control and Prevention, Tongshan 221006, China; 9 Jonsson Comprehensive Cancer Center, UCLA, Los Angeles, CA 90095, USA; 10 School of Nursing, UCLA, Los Angeles, CA 90095, USA; 11 Department of Epidemiology and Environmental Health, School of Public Health and Health Professions, University at Buffalo, The State University of New York, Buffalo, NY 14214, USA; 12 Center for Human Nutrition, Department of Medicine, UCLA David Geffen School of Medicine, Los Angeles, CA 90095, USA; 13 Department of Epidemiology, School of Public Health, Peking University, Beijing 100083, China

**Keywords:** liver cancer, garlic, hepatitis B virus, interaction, Chinese population

## Abstract

Although the major risk factors for liver cancer have been established, preventive factors for liver cancer have not been fully explored. We evaluated the association between raw garlic consumption and liver cancer in a large population-based case-control study in Eastern China. The study was conducted in Jiangsu, China, from 2003 to 2010. A total of 2011 incident liver cancer cases and 7933 randomly selected population-controls were interviewed. Epidemiological data including raw garlic intake and other exposures were collected, and serum markers of hepatitis B virus (HBV) and hepatitis C virus (HCV) infection were assayed. Overall, eating raw garlic twice or more per week was inversely associated with liver cancer, with an adjusted odds ratio (aOR) of 0.77 (95% confidence interval (CI): 0.62–0.96) compared to those ingesting no raw garlic or less than twice per week. In stratified analyses, high intake of raw garlic was inversely associated with liver cancer among Hepatitis B surface antigen (HBsAg) negative individuals, frequent alcohol drinkers, those having history of eating mold-contaminated food or drinking raw water, and those without family history of liver cancer. Marginal interactions on an additive scale were observed between low raw garlic intake and HBsAg positivity (attributable proportion due to interaction (AP) = 0.31, 95% CI: -0.01–0.62) and heavy alcohol drinking (AP = 0.28, 95% CI: 0.00–0.57). Raw garlic consumption is inversely associated with liver cancer. Such an association shed some light on the potential etiologic role of garlic intake on liver cancer, which in turn might provide a possible dietary intervention to reduce liver cancer in Chinese population.

## 1. Introduction

It was estimated that 841,000 new patients with liver cancer were diagnosed worldwide in 2018, and 46.7% of them occurred in China [1]. Liver cancer is a highly lethal cancer with an overall five-year survival rate of 18.4%, ranging from 2.4% (distant) to 32.6% (localized) depending on stage at diagnosis [2]. Only a small proportion of liver cancer patients are detected at an early stage for curative therapies, such as surgical resection or liver transplantation. Although risk factors, including infections of hepatitis B virus (HBV) and hepatitis C virus (HCV), alcohol consumption, liver cirrhosis, dietary aflatoxins, and tobacco smoking have been established, little is known about the protective factors for liver cancer [3,4]. Thus, it is of importance to explore potential protective factors for early intervention of the disease.

*Allium sativum* (garlic) is a species in the onion genus, *Allium*. It has been used by humans in both culinary and traditional medicine for thousands of years worldwide. There have been research interests in the anti-carcinogenic effect of garlic. The activity of isolated garlic constituents has been studied in experimental studies. The organosulfur compounds (OSCs), including diallyl sulfide (DAS), diallyl disulfide (DADS), diallyl trisulfide (DATS) and ajoene from garlic, were found to have anti-cancer effects in experimental studies [5,6]. A population-based cohort study in China showed an inverse association between habitual garlic consumption and all-cause mortality [7]. Furthermore, the associations of garlic intake with cancer risks have been evaluated for a variety of sites, including stomach [8,9,10,11,12,13,14,15], colon and rectum [16,17,18,19], esophagus [15,20,21,22], breast [23,24,25,26], lung [27,28], larynx [29] and prostate [30,31,32,33,34]. Most of these studies reported inverse associations. However, to the best of our knowledge, there has been no epidemiologic study published on the association of raw garlic intake with liver cancer in the medical literature.

We investigated the association of raw garlic consumption with liver cancer in a population-based case-control study in four counties of Jiangsu Province, China, and further explored the potential interactions between raw garlic intake and known risk factors on the development of liver cancer.

## 2. Materials and Methods

### 2.1. Study Design

We conducted a population-based case-control study in four counties of Jiangsu Province, China, including Dafeng, Ganyu, Chuzhou and Tongshan from 2003 to 2010. The study design and methods were described in detail in a previous publication [35]. Briefly, four parallel, individually-matched case-control studies by age, gender and residence were conducted on cancers of the esophagus, stomach, liver and lung. In the data analyses for liver cancer, controls from all four cancer sites were pooled as controls to increase the power of the study.

### 2.2. Subjects

Liver cancer patients were identified from respective county cancer registries during 2003 to 2010. The inclusion criteria for the cases were: (1) Aged 18 years or above; (2) had resided in the county for at least 5 years; (3) newly diagnosed as primary liver cancer within 12 months. Healthy population controls were randomly selected and 1:1 matched to cases by age (±5 years) and gender from the same county. People with a history of any cancer were excluded from the study. The response rates were 37% among liver cancer cases and 87% among all controls.

### 2.3. Data Collection

The study was approved by the Institutional Review Board (IRB) of Jiangsu Provincial Health Department and the IRB of University of California, Los Angeles. After written informed consent obtained from each participant, a face-to-face interview was carried out by trained interviewers using a standard questionnaire. Epidemiologic data on the questionnaire include: (1) Socio-demographic characteristics, including age, gender, level of education, marital status and family income 10 years ago, and weight and height were measured at the interview; (2) weekly raw garlic consumption. Participants were asked if they consumed raw garlic every week in their lifetime. The options for answer included: Never, less than twice per week and twice or more per week; (3) exposure to known risk factors of liver cancer and potential confounding factors, including alcohol consumption, tobacco smoking, a history of eating mold-contaminated food (as surrogates for aflatoxin contamination), a history of drinking raw water (drank water directly from ditch, river, well, tap, etc., without boiling or purification; it was probably contaminated by pesticides, microorganisms or microcystin, used here as surrogates for unsafe water intake), family history of liver cancer, etc. Ever smokers were defined as those have smoked more than 100 cigarettes lifetime. For ever-smokers, number of cigarettes smoked every day and total years of smoking were asked, and pack-year of smoking was calculated. Alcohol drinking frequency was asked, and options for answer included never (never drinks alcohol, including those only drink a little on holidays), occasionally (less than twice per week), drinking frequently (2 to 6 times per week) and drinking every day. Those who drank at a frequency of occasionally or higher were considered as ever drinkers. The type and amount of alcohol consumed was asked and calculated as weekly consumption of ethanol in milliliter. Blood samples were collected after the questionnaire interviews. Serum HBsAg, Hepatitis B surface antibody (HBsAb), Hepatitis B e antigen (HBeAg), Hepatitis B e antibody (HBeAb), Hepatitis B core antibody (HBcAb) and anti-HCV antibody were measured at Jiangsu Provincial Center for Disease Control and Prevention (CDC) using an enzyme-linked immunosorbent assay (ELISA) (Shanghai Kehua Diagnostic Medical Products Co., Ltd., Shanghai, China) according to the manufacturer’s protocol.

### 2.4. Statistical Analyses

Statistical analyses were performed using the SAS 9.3 package (SAS Institute Inc., Cary, NC, USA). Chi-squared tests were used to compare categorical variables. Since individual matching was broken, unconditional logistic regression models were used to estimate crude odds ratios (cOR) and adjusted odd ratios (aOR) with their 95% confidence intervals. The following potential confounding factors were adjusted for in multiple logistic regression: Age (continuous), gender (male = 1, female = 0), level of education (illiteracy = 0, primary school = 1, middle school = 2, high school and college = 3, as dummy variables), marital status (in marriage = 1, not in marriage = 0), family income 10 years ago per capita (RMB Yuan/year, continuous), body mass index (BMI, continuous), having a family history of liver cancer (yes = 1, no = 0), smoking (pack-year, continuous), ethanol consumption (mL/week, continuous), history of eating mold-contaminated food (yes = 1, no = 0), history of drinking raw water (yes = 1, no = 0), HBsAg status (positive = 1, negative = 0), anti-HCV status (positive = 1, negative = 0) and study area (Dafeng = 1, Ganyu = 2, Chuzhou = 3, Tongshan = 4, as dummy variables).

Potential additive and multiplicative interactions were assessed by relative excess risk due to interaction (RERI), attributable proportion due to interaction (AP), synergy index (S) and ratio of the odds ratios (ROR), adjusting for potential confounding factors [36]. In the analyses for interactions, preventive factors were re-coded and the stratum with the lowest risk was set as the reference category for examining the joint effect.

A semi-Bayes (SB) method was used to reduce the chance for potential false positive findings by multiple comparisons using null priors (OR = 1.00, 95% prior limits: 0.25, 4.00), corresponding to the coefficients of mean zero and variance of 0.5 [37].

## 3. Results

A total of 2011 incident liver cancer cases and 7933 controls were included in the study. Since the controls were combined from four parallel studies, liver cancer cases and the controls significantly differed by age and gender, which is largely due to the different age- and gender-distributions of the cases from four cancer sites (liver, lung, stomach and esophagus). We also observed the differences between cases and controls by BMI, level of education, marital status and proportion of having family history of liver cancer, except per capita family income 10 years ago. The prevalence of raw garlic consumption by socio-demographic characteristics, along with major risk factors of liver cancer, including alcohol consumption, tobacco smoking and family history of liver cancer, are presented by the cases and the controls in Table 1.

The associations of raw garlic intake with liver cancer are shown in Table 2. Overall, 12.8% of liver cancer cases and 15.5% of controls reported to ingest raw garlic twice or more per week. After adjusting for potential confounding variables, raw garlic consumption less than twice per week was not significantly associated with liver cancer compared to those who never consumed garlic (aOR: 1.02, 95% CI: 0.84–1.23), while consuming raw garlic twice or more per week was inversely associated with an aOR of 0.78 (95% CI: 0.62–1.01). Comparing to the reference group (individuals who consume less than twice a week or never ingest raw garlic), the aOR was 0.77 (95% CI: 0.62–0.96) for raw garlic consumption twice or more per week. Furthermore, the point estimate remained the same magnitude after semi-Bayes adjustment.

Stratified analyses for associations of consumption of raw garlic with liver cancer by major risk factors and potential confounders are shown in Table 3. Comparing intake of raw garlic twice or more per week to the reference group for liver cancer, the aOR was 0.68 (95% CI: 0.53–0.87) in Tongshan, which was the county with the largest sample size among the four counties. Among those HBsAg-negative participants, raw garlic consumption was inversely associated with liver cancer (aOR: 0.69, 95% CI: 0.54–0.88). Among those frequent alcohol drinkers defined as drinking alcohol frequently or daily, consumption of raw garlic twice or more per week was inversely associated with liver cancer with an aOR of 0.41 (95% CI: 0.26–0.66). Inverse associations were also observed among those who had a history of drinking raw water (aOR: 0.70, 95% CI: 0.52–0.94), those who had a history of eating mold-contaminated food (aOR: 0.37, 95% CI: 0.17–0.81) and those who did not have a family history of liver cancer (aOR: 0.77, 95% CI: 0.61–0.96). Semi-Bayes estimates showed similar associations in the stratified analyses.

Potential joint effects between low garlic consumption (garlic consumption < twice per week) and other risk factors were analyzed (Table 4). Compared to those HBsAg negative participants who had raw garlic intake ≥twice/week, those HBsAg negative with low garlic consumption had an aOR of 1.25 (95% CI: 0.98–1.60); those HBsAg positive with high garlic consumption had an aOR of 8.53 (95% CI: 5.26–13.83), while those HBsAg positive with low garlic consumption had an aOR of 12.67 (95% CI: 9.54–16.83). After adjusting for potential confounders, marginally significant interaction on an additive scale was observed between low garlic consumption and HBsAg positivity (AP = 0.31, 95% CI: −0.01–0.62), and heavy alcohol drinking (AP = 0.28, 95% CI: 0.00–0.57). No obvious multiplicative interactions were observed between low garlic intake with risk factors on liver cancer.

## 4. Discussion

It is the first time in the published medical literature, that an inverse association of raw garlic intake with liver cancer has been observed from a large population-based case-control study in China. After controlling for known risk factors and potential confounders, frequent consumption of raw garlic (more than twice per week) showed an inverse association, which suggests the potential preventive effect of raw garlic intake. Potential additive interactions were also identified between low raw garlic intake and HBV infection or heavy alcohol drinking.

The adjusted odds ratio and its 95% confidence interval for raw garlic consumption were 0.77 (95% CI: 0.62–0.96) suggesting that raw garlic intake twice or more per week may have a preventive effect on liver cancer. The results from epidemiologic studies on garlic intake and risk of other cancers remained inconsistent. Although many identified a protective effect [8,9,10,11,12,13,15,17,18,22,24,28,29,30,31,32,33], several other studies reported a null association [14,16,19,23,25,34]. In these studies, some groups examined allium vegetables, including garlic, onions and leeks. Most previously published studies had relatively small sample sizes and inadequately adjusted for potential confounding factors. The method of preparation or processing of garlic also varied from raw or cooked, to extracts. Preparation of garlic may have an impact on its bioavailability and anti-carcinogenic effect. We collected data on raw garlic intake frequency, firstly because we would like to examine the effects of the constituents as a whole, and those constituents are mostly the metabolites of allicin, which is abundant in fresh garlic. Secondly, in these study-areas of China, people usually ingest raw garlic cloves directly, unlike other areas, where garlic is typically cooked. This is most common in the Ganyu and Tongshan Districts in the Northern Jiangsu Province where people have a habit of the consumption of raw garlic directly [11]. This dietary habit is relatively specific to this region, and made it possible for us to examine the association between raw garlic intake and liver cancer with a large sample size.

Experimental studies have shown preventive effects of garlic, as well as its isolated constituents on cancer development through different molecular mechanisms [38,39,40,41,42,43,44,45]. These mechanisms may include modulation of metabolizing enzymes, such as cytochrome P450s and glutathione S-transferases, which may have implications for the deactivation or detoxification of carcinogens, suppression of the formation of DNA adducts, induction of cell-cycle arrest, apoptosis, cell differentiation and cell invasion [5,6]. For the first time, our study provides the human epidemiological evidence on the potential protective effect of raw garlic on liver cancer.

In addition to the main effect of raw garlic intake on the development of liver cancer, potential effect measure modifications were analyzed on both additive and multiplicative scales between garlic intake and other risk factors. Departures from additivity have been observed between raw garlic intakes with HBsAg status and heavy alcohol drinking. This might suggest a possible biological interaction for the underlying biological mechanisms of protection by garlic on liver injury induced by those exposures, which had been speculated in some animal studies [46,47]. In stratified analyses, inverse associations of raw garlic intake with liver cancer were observed, especially among participants from Tongshan, males, those HBsAg negative participants, frequent alcohol drinkers, those who might have been exposed to aflatoxin and those who did not have family history of liver cancer. As above mentioned, people in Ganyu and Tongshan consumed raw garlic more frequently, compared to the other two counties (Table 1). The inverse associations were observed in three counties, but not in Dafeng because of very low frequency of garlic consumption. A significant association was mainly observed in Tongshan, which had the highest proportion of the garlic intake and a larger sample size. According to GLOGOBCAN 2018, men had higher incidence of liver cancer than women, both worldwide (13.9 versus 4.9 per 100, 000) and in China (27.6 versus 9.0 per 100,000) [1]. Raw garlic intake only showed protective potential in males, probably because men had more frequent intake (17.0% versus 11.5% among controls) and a larger sample size (more than 70% of the study population). The inverse associations of frequent ingestion of raw garlic with liver cancer were only observed among HBsAg negative participants, but not HBsAg positive individuals in our study. It is possible that the damage to liver cells caused by long-term inflammation and irregular regeneration are not reversible, as HBV infection is a dominant risk factor for liver cancer. Raw garlic intake might not be effective in reversing the severe liver damage induced by HBV. Nevertheless, since most of the population is HBV free, especially in younger generations with HBV vaccine coverage, frequent raw garlic intake would provide wider protections against liver cancer. Moreover, observed inverse associations of raw garlic intake with liver cancer, among frequent alcohol drinkers, those had history of drinking raw water or eating mold-contaminated food in this study, further suggest protective benefits of raw garlic for people with known risk factors.

All cases in this study were newly diagnosed. They were identified and recruited through population-based cancer registries in four counties. Random samples of healthy individuals were selected from the source population as controls. However, because of the short survival of liver cancer patients, the participation rate was relatively low for cases in this study. We tried our best to reach out to patients once the diagnoses were reported to the cancer registries, and conducted the interviews at their homes. However, due to an inevitable time lag from report to recruitment, the cases enrolled and those who had blood drawn might be the ones with less advanced stages, or had less exposure to known risk factors. As a result, selection bias might have had some impact on the observed associations, and might limit the generalizability of the study results to general population.

Information bias may also exist in the measurement of the exposures and confounding factors because of the difficulty and imprecision with the retrospective recalling of behaviors from long ago. Since raw garlic intake was not recognized as a protective or risk factor for liver cancer, the possibility of recall bias of exposure might be minimal. In order to increase the validity of our data collected, we provided two-day intensive training sessions and field practices for interviewers, annually, to improve the adherence to the study protocol. Just like other risk factors, such as tobacco smoking, alcohol drinking and other exposures in epidemiological studies, it is very difficult to validate these self-reported risk factors, including garlic intake. However, a random sample of 10% of the subjects were re-interviewed for selected risk factors, and showed an overall accuracy over 95% for both cases and controls [35]. In addition, we could not assess or compare different processes in preparation of garlic (raw, pickled or cooked), which limited our ability to study the associations of cooked garlic, garlic powder, or garlic isolates’ intakes with liver cancer. Future studies should also collect the data on methods of garlic preparation, which might gain additional value in the study of garlic in relation to liver cancer.

Although we tried to measure and include major known risk factors of liver cancer in this study, including a history of mold-contaminated food and raw water intake as proxies for aflatoxin and other toxins, other potential confounding factors, including dietary factors or patterns, nutritional status, and other liver diseases, were not adjusted for in this study. We conducted sensitivity analyses, including both fatty liver disease and liver cirrhosis, in the multivariable logistic regression model; the estimate for eating raw garlic twice or more per week was 0.77 (95% CI: 0.62–0.97) compared to less or never takers, which was almost identical to the current results. Meanwhile, since the impact of other nutritional factors on liver cancer remained largely unknown, with an exception of dietary Aflatoxin B1, the residual confounding effects by other nutritional factors might be minor in distorting the observed association. In this study, we adjusted for mold-contaminated foods, so that the potential confounding effects by other nutritional factors would be minimized. In addition, we are planning to analyze the data to explore potential impacts of other dietary factors in our future studies.

In conclusion, an inverse association was observed between raw garlic consumption and liver cancer in a Chinese population. Possible additive interactions were suggested between raw garlic intake with HBV infection, as well as heavy alcohol drinking. These findings suggest a potential role of raw garlic’s intake on the development of liver cancer and might potentially serve as an intervention agent for chemoprevention to reduce incidence of liver cancer. Intake of raw garlic is relatively popular in the study area. Many studies have been conducted in the world, including ones in Italy, Serbia, Turkey, Netherlands, France, Iran, Puerto Rico, etc., on the associations of garlic intake and cancer, indicating a great research interest in etiology and prevention. Our results may add some epidemiological evidence to the potential preventive role of garlic, and appeal for further exploration into the biologically active constituents of raw garlic and the underlying mechanism. If the preventive effect could be confirmed in the future studies, we would recommend to have more raw garlic on dining tables, or to develop products with effective extracts from raw garlic.

## Figures and Tables

**Table 1:** Socio-demographic characteristics of subjects in Jiangsu liver cancer study (2003–2010).

Characteristics	Case				Control			
	Number (Proportion %)	Raw Garlic Consumption (%)			Number (Proportion %)	Raw Garlic Consumption (%)		
		Never	<Twice/Week	≥Twice/Week		Never	<Twice/Week	≥Twice/Week
Total	2011	45.5	41.7	12.8	7933	46.0	38.6	15.5
County of residence				*p* < 0.001				*p* < 0.001
Dafeng	632 (31.4)	76.6	20.5	2.9	2534 (31.9)	77.0	20.6	2.3
Ganyu	390 (19.4)	21.9	62.9	15.2	2010 (25.3)	23.4	55.5	21.1
Chuzhou	301 (15.0)	67.7	29.3	3.0	1134 (14.3)	69.3	25.8	4.9
Tongshan	688 (34.2)	20.5	54.6	24.9	2255 (28.4)	19.8	49.9	30.2
Gender				*p* = 0.001				*p* < 0.001
Male	1534 (76.3)	43.6	42.3	14.1	5705 (71.9)	42.2	40.8	17.0
Female	477 (23.7)	51.6	39.7	8.7	2228 (28.1)	55.5	33.1	11.5
Age group (years)				*p* = 0.228				*p* < 0.001
<50	471 (23.4)	47.7	39.1	13.3	872 (11.0)	43.3	39.4	17.4
50–	603 (30.0)	44.9	40.9	14.2	1773 (22.4)	42.5	39.4	18.2
60–	515 (25.6)	45.3	43.1	11.6	2542 (32.0)	49.1	36.4	14.5
70–	322 (16.0)	47.4	42.1	10.6	2168 (27.3)	45.1	40.8	14.2
80–	100 (5.0)	33.3	50.5	16.2	578 (7.3)	49.9	36.7	13.3
Marital Status				*p* = 0.570				*p* < 0.001
In marriage	1717 (86.0)	45.0	42.0	13.1	6426 (81.5)	44.2	39.6	16.2
Single, divorced or widowed	279 (14.0)	48.2	40.2	11.6	1463 (18.5)	53.5	34.5	12.1
Level of education				*p* = 0.021				*p* < 0.001
Illiteracy	764 (38.2)	45.3	44.7	10.0	3796 (48.0)	47.5	38.8	13.7
Primary school	662 (33.1)	43.8	42.0	14.3	2490 (31.5)	46.4	37.5	16.2
Middle school	461 (23.1)	46.4	38.7	14.9	1298 (16.4)	39.7	40.3	20.0
High school	101 (5.1)	55.5	29.7	14.9	292 (3.7)	50.3	38.6	11.0
College	12 (0.6)	27.3	54.6	18.2	40 (0.5)	42.5	37.5	20.0
Per capita family income 10 years ago (RMB yuan/year)				*p* < 0.001				*p* < 0.001
<1000	392 (20.0)	37.4	47.2	15.4	1656 (21.3)	36.5	44.7	18.8
1000–	405 (20.6)	40.5	46.4	13.2	1512 (19.5)	41.4	42.6	16.0
1500–	487 (24.8)	46.6	41.0	12.4	2057 (26.5)	48.0	37.0	15.0
2500–	680 (34.6)	51.3	37.0	11.7	2542 (32.7)	52.9	33.7	13.4
BMI (kg/m^2^)				*p* = 0.341				*p* = 0.015
<23.0	1290 (65.2)	47.5	41.1	11.4	4185 (53.0)	46.4	38.0	15.6
23.0–	603 (30.5)	42.1	42.5	15.4	3126 (39.6)	44.6	40.0	15.5
27.5–	56 (2.8)	42.9	46.4	10.7	474 (6.0)	53.0	33.3	13.7
32.5–	7 (0.4)	42.9	42.9	14.3	25 (0.3)	60.0	32.0	8.0
37.5–	24 (1.2)	47.8	43.5	8.7	80 (1.0)	35.0	43.8	21.3
Ever smoked tobacco (more than 100 cigarettes lifetime)				*p* = 0.327				*p* < 0.001
No	971 (48.3)	43.8	43.3	13.0	4241 (53.5)	45.4	37.8	16.9
Yes	1040 (51.7)	47.0	40.3	12.7	3692 (46.5)	46.6	39.6	13.8
Ever drunk alcohol (occasionally or more frequently)				*p* < 0.001				*p* = 0.029
No	885 (44.0)	52.0	38.1	10.0	4254 (53.6)	46.8	37.3	16.0
Yes	1126 (56.0)	40.4	44.5	15.1	3679 (46.4)	45.0	40.2	14.9
Have family history of liver cancer				*p* < 0.001				*p* < 0.001
No	1735 (86.3)	43.1	43.2	13.8	7684 (96.9)	45.2	39.0	15.8
Yes	276 (13.7)	60.5	32.6	6.9	249 (3.1)	69.4	25.0	5.7
HBsAg positive				*p* < 0.001				*p* = 0.213
No	689 (56.8)	35.9	45.6	18.5	6074 (93.5)	46.6	38.4	15.0
Yes	524 (43.2)	51.3	37.0	11.7	425 (6.5)	48.9	39.2	11.9

**Table 2:** The association and semi-Bayes (SB) adjusted association of raw garlic consumption with liver cancer in the Jiangsu Study (2003–2010).

Variables	Case *n* = 2011	%	Control *n* = 7933	%	Crude		Adjusted ^a^		SB-Adjusted ^b^	
					OR	95% CI	OR	95% CI	OR	95% PI
Raw garlic consumption										
Never	907	45.5	3628	46.0	1.00		1.00		1.00	
<twice a week	832	41.7	3048	38.6	1.09	0.98–1.21	1.02	0.84–1.23	1.02	0.84–1.23
≥Twice a week	256	12.8	1220	15.5	0.84	0.72–0.98	0.78	0.62–1.01	0.79	0.62–1.00
*p*-trend					0.234		0.100			
Raw garlic consumption										
Never or <twice/week	1739	87.2	6676	84.6	1.00		1.00		1.00	
≥Twice a week	256	12.8	1220	15.5	0.81	0.70–0.93	0.77	0.62–0.96	0.77	0.62–0.96

OR, odds ratio; CI, confidence interval; SB, semi-Bayes adjustment; PI, posterior interval; HBsAg, Hepatitis B surface antigen. ^a^ Adjusted for age (continuous), gender (male = 1, female = 0), education level (illiteracy = 0, primary school = 1, middle school = 2, high school or college = 3), married (yes = 1, no = 0), family income 10 years ago per capita (RMB Yuan/year continuous), BMI (continuous), having a family history of liver cancer (yes = 1, no = 0), pack-year of smoking (continuous), ethanol consumption (mL/week, continuous), history of eating mold-contaminated food (yes = 1, no = 0), history of drinking raw water (yes = 1, no = 0), HBsAg status (positive = 1, negative = 0), anti-HCV (positive = 1, negative = 0) and study area (Dafeng = 1, Ganyu = 2, Chuzhou = 3, Tongshan = 4). ^b^ Semi-Bayes adjustment using prior: 1.00, 95% CI: 0.25, 4.00.

**Table 3:** The association and SB-adjusted association of raw garlic consumption with liver cancer stratified by major risk factors in the Jiangsu Study (2003–2010).

Variables for Stratification	Raw Garlic Consumption ≥ Twice per Week			
	Adjusted ^a^		SB-Adjusted ^b^	
	OR	95% CI	OR	95% PI
All	0.77	0.62–0.96	0.77	0.62–0.96
Study area				
Dafeng	1.33	0.55–3.20	1.23	0.58–2.58
Ganyu	0.72	0.37–1.41	0.77	0.42–1.40
Chuzhou	0.57	0.10–3.33	0.81	0.27–2.39
Tongshan	0.68	0.53–0.87	0.69	0.54–0.88
Gender				
Male	0.73	0.57–0.94	0.74	0.58–0.94
Female	0.89	0.55–1.42	0.90	0.58–1.41
HBsAg status				
Negative	0.69	0.54–0.88	0.70	0.55–0.89
Positive	0.93	0.57–1.53	0.94	0.59–1.49
Alcohol drink				
Never or <twice per week	0.92	0.71–1.19	0.92	0.72–1.19
≥twice per week	0.41	0.26–0.66	0.45	0.29–0.70
Tobacco smoke				
Never	0.84	0.63–1.11	0.85	0.64–1.12
Ever	0.76	0.55–1.06	0.77	0.56–1.06
History of drinking raw water				
No	0.89	0.63–1.26	0.90	0.64–1.25
Yes	0.70	0.52–0.94	0.71	0.53–0.95
History of eating mold-contaminated food				
No	0.82	0.65–1.04	0.82	0.65–1.04
Yes	0.37	0.17–0.81	0.47	0.24–0.93
Family history of liver cancer				
No	0.77	0.61–0.96	0.78	0.62–0.97
Yes	0.87	0.21–3.67	0.93	0.35–2.53

OR, odds ratio; CI, confidence interval; SB, semi-Bayes adjustment; PI, posterior interval; HBsAg, Hepatitis B surface antigen. ^a^ Adjusted for age (continuous), gender (male = 1, female = 0), education level (illiteracy = 0, primary school = 1, middle school = 2, high school or college = 3), in a marriage (yes = 1, no = 0), family income 10 years ago per capita (RMB Yuan/year continuous), BMI (continuous), family history of liver cancer (yes = 1, no = 0), pack-year of smoking (continuous), ethanol consumption (mL/week, continuous), history of eating mold-contaminated food (yes = 1, no = 0), history of drinking raw water (yes = 1, no = 0), HBsAg status (positive = 1, negative = 0), anti-HCV status (positive = 1, negative = 0) and study area (Dafeng = 1, Ganyu = 2, Chuzhou = 3, Tongshan = 4), except when stratified by each variable. ^b^ semi-Bayes adjustment using prior: 1.00 (95% CI: 0.25, 4.00).

**Table 4:** The effect modification between raw garlic consumption and other risk factors for liver cancer in the Jiangsu Study (2003–2010).

Risk Factor	Raw Garlic Intake ≥ Twice/Week	Case	Control	Crude		Adjusted ^a^		Interaction
				OR	95% CI	OR	95% CI	
HBsAg positive								
No	Yes	127	907	1.00		1.00		RERI: 3.88 (−0.49–8.25)
No	No	561	5144	0.78	0.63–0.96	1.25	0.98–1.60	AP: 0.31 (−0.01–0.62)
Yes	Yes	61	50	8.71	5.74–13.23	8.53	5.26–13.83	S: 1.5 (0.90–2.50)
Yes	No	461	371	8.87	7.04–11.17	12.67	9.54–16.83	ROR: 1.18 (0.71–1.99)
Anti-HCV status								
No	Yes	185	944	1.00		1.00		RERI: −2.97 (−9.03–3.09)
No	No	1016	5475	0.95	0.80–1.12	1.32	1.06–1.65	AP: −2.27 (−7.75–3.20)
Yes	Yes	3	10	1.53	0.42–5.62	3.95	0.88–17.74	S: 0.09 (0.00–8.21)
Yes	No	8	43	0.95	0.44–2.05	1.30	0.49–3.44	ROR: 0.25 (0.04–1.48)
Heavy alcohol drinking								
No	Yes	165	889	1.00		1.00		RERI: 0.6 (−0.02–1.22)
No	No	1113	4795	1.25	1.05–1.50	1.18	0.91–1.53	AP: 0.28 (0.00–0.57)
Yes	Yes	91	331	1.48	1.11–1.97	1.34	0.86–2.09	S: 2.14 (0.63–7.22)
Yes	No	626	1881	1.79	1.48–2.17	2.12	1.57–2.86	ROR: 1.34 (0.83–2.14)
Ever smoked tobacco								
No	Yes	124	712	1.00		1.00		RERI: 0.06 (−0.47–0.60)
No	No	833	3507	1.36	1.11–1.67	1.26	0.96–1.66	AP: 0.04 (−0.27–0.34)
Yes	Yes	132	508	1.49	1.14–1.95	1.46	1.00–2.13	S: 1.09 (0.51–2.33)
Yes	No	906	3169	1.64	1.34–2.01	1.78	1.33–2.39	ROR: 0.97 (0.65–1.46)
History of eating mold-contaminated food ^b^								
No	Yes	222	1096	1.00		1.00		RERI: 0.11 (−0.53–0.74)
No	No	1533	6098	1.24	1.06–1.45	1.22	0.97–1.55	AP: 0.06 (−0.30–0.43)
Yes	Yes	31	111	1.38	0.90–2.11	0.73	0.37–1.43	S: 1.17 (0.43–3.23)
Yes	No	186	532	1.73	1.39–2.15	1.60	1.12–2.27	ROR: 1.78 (0.86–3.70)
History of drinking raw water								
No	Yes	91	597	1.00		1.00		RERI: 0.05 (−0.48–0.57)
No	No	616	2920	1.38	1.09–1.75	1.33	0.95–1.87	AP: 0.03 (−0.27–0.33)
Yes	Yes	161	605	1.75	1.32–2.31	1.38	0.93–2.05	S: 1.07 (0.50–2.28)
Yes	No	1073	3601	1.96	1.55–2.46	1.76	1.26–2.46	ROR: 0.96 (0.62–1.47)
Family history of liver cancer								
No	Yes	237	1206	1.00		1.00		RERI: −0.73 (−7.79–6.34)
No	No	1482	6442	1.17	1.01–1.36	1.31	1.05–1.64	AP: −0.13 (−1.42–1.16)
Yes	Yes	19	14	6.90	3.41–13.96	5.93	1.82–19.30	S: 0.86 (0.22–3.35)
Yes	No	257	234	5.59	4.46–7.00	5.51	3.85–7.90	ROR: 0.71 (0.21–2.38)

OR, odds ratio; CI, confidence interval; SB, semi-Bayes adjustment; PI, posterior interval; HBsAg, Hepatitis B surface antigen; RERI, relative excess risk due to interaction; AP, attributable proportion due to interaction; S, synergy index; ROR, ratio of odds ratio. ^a^ Adjusted for age (continuous), gender (male = 1, female = 0), education level (illiteracy = 0, primary school = 1, middle school = 2, high school or college = 3), in a marriage (yes = 1, no = 0), family income 10 years ago per capita (RMB Yuan/year continuous), BMI (continuous), having a family history of liver cancer (yes = 1, no = 0), pack-year of smoking (continuous), ethanol consumption (mL/week, continuous), history of eating mold-contaminated food intake (yes = 1, no = 0), history of drinking raw water (yes = 1, no = 0), HBsAg status (positive = 1, negative = 0), anti-HCV status (positive = 1, negative = 0) and study area (Dafeng = 1, Ganyu = 2, Chuzhou = 3, Tongshan = 4), except when the variable was examined. ^b^ The interaction between less or never raw garlic intake and history of eating mold-contaminated food was examined in the crude model without adjusting for potential confounders, because the directions of the associations comparing the three strata to the reference group were different in the adjusted model.
